# A call for a novel and next-generation vaccine against monkeypox disease

**DOI:** 10.1016/j.amsu.2022.104968

**Published:** 2022-11-17

**Authors:** Manojit Bhattacharya, Kuldeep Dhama, Chiranjib Chakraborty

**Affiliations:** Department of Zoology, Fakir Mohan University, Vyasa Vihar, Balasore, 756020, Odisha, India; Division of Pathology, ICAR-Indian Veterinary Research Institute, Izatnagar, Bareilly, 243122, Uttar Pradesh, India; Department of Biotechnology, School of Life Science and Biotechnology, Adamas University, Kolkata, West Bengal, 700126, India

Dear Editor,

The monkeypox virus (MPXV) continues to spread across different countries in the world. The disease caused by MPXV is an uncommon viral zoonotic disease that is occasionally considered life-threatening. Earlier, the monkeypox disease was epidemic to the African countries, and it spread later to various populations and numbers of geographical areas of non-African countries. Genomic instability and mutational changes also become the significant factors for the re-emergence of human MPXV, animals (non-human) to human, and human to human transmission [1,2].

Several countries (e.g. USA, UK, Canada) have initiated a “ring vaccination” strategy to resist the spread of MPXV, which is a selective vaccination approach that successfully contained smallpox and Ebola outbreaks. The Centers for Disease Control and Prevention (CDC) advises people who are considered at high risk for MPXV infection to get vaccinated against the virus [3]. But precisely, the vaccine candidate has not been developed specifically against MPXV, and therefore smallpox vaccines are recommended assuming to be 85% effective by providing cross-protective immunity against the MPXV infection, as per WHO and CDC. However, smallpox vaccines have minimal testing against MPXV infection, and this assessment was based on the past data from the infection in the African region, where the MPXV outbreaks occurred decades ago [4].

Currently, some vaccines being used against smallpox infection have also been applied to prevent MPXV infection in humans (Table 1) [5]. Out of these vaccines, two vaccines were licensed for use against infection in the USA, JYNNEOS (known as Imvanex or Imvamune) and ACAM200 [6]. The JYNNEOS is a live non-replicating virus vaccine, which is given as two injections within four weeks [7], while the ACAM2000 vaccine is given as a live virus preparation by pricking the skin surface that grows at the lesion site and might spread to other parts of the body or other people. Having questions about the side effects of MPX vaccines is reasonable. The JYNNEOS vaccine has few side effects after vaccination, such as headache, fatigue, nausea, pain at the injection spot, swelling, and redness visible at the injection site within the first couple of days after vaccination [5]. In the ACAM2000 vaccination, individuals suffered fever, small rash, lymph node swelling, and other associated adverse complications (myocarditis and pericarditis) along with pain, swelling, and redness. This vaccine is unsafe and risky for pregnant women, infants, immunocompromised, or individuals living with HIV [8]. The JYNNEOS vaccine is considered to be less reactogenic rather than other traditional smallpox vaccines. Another specialized vaccine known as Aventis Pasteur Smallpox Vaccine (APSV) is presently being offered to humans in a few countries (UK, Spain) [9].

**Table 1:** Smallpox vaccines and their status, used against the MPXV infection.

Sl. No.	Vaccine name	Category	Status	Types
1.	JYNNEOS™ (Imvamune or Imvanex or MVA-BN)	Recent-advanced smallpox vaccines	Approved for smallpox and human MPXV	Modified Vaccinia Ankara-Bavarian Nordic (MVA-BN) strain, attenuated Vaccinia virus,
2.	ACAM2000®			Live vaccinia virus (replication competent)
3.	Aventis Pasteur Smallpox Vaccine (APSV)	Old smallpox vaccines	Under Investigational New Drug Application [IND] or Emergency Use Authorization (EUA) for smallpox emergency	Replication-competent of vaccinia virus
4.	Modified vaccinia virus Ankara (MVA)		Licensed as third-generation vaccine against smallpox	Non-replicative, highly attenuated modified vaccinia virus Ankara (MVA) strain
5.	VACV Lister strain (VACV-LST)		Most widely used as smallpox vaccine	Replicative, attenuated vaccinia virus
6.	New York City Board of Health (NYCBOH) (Dryvax)		Discontinued in 1982	Replicative, multiclonal vaccinia vaccine
7.	Western Reserve		Consider as model virus for most studies on aspects of poxvirus biology	Replicative, vacinia strain

Of note, a specialized next-generation vaccine can be developed based on the genomic sequence information of MPXV. The essential viral protein(s) is considered as vaccine antigens and used as the coding sequence for vaccine development. This peptide-based vaccine formulation platform is highly acceptable rather the conventional procedure of vaccine development. It should have higher efficiency, be less expensive, and speed up the process of covering different mutational variants of this virus [10]. Most nucleic acid-based vaccines, such as DNA and mRNA, can quickly be adapted in case of a newly emerging virus involving a next-generation vaccine development platform.

Next-generation vaccine should be the most reliable vaccine platform for manufacturing vaccine candidates in a faster way when a new pathogenic virus emerges shortly and acquire mutations. If we look at the evolution of the MPXV, the virus separated from Orthopoxviruses approximately 3500 years ago through the continuous evolution derived from the mutation. It has been observed that undergoing the evolution process, and the genetic variation produced the MPXV West African subtype about 600 years ago. Researchers have also informed about two initial clades of the African MPXV, which are i) CB Clade or the Congo Basin Clade (Clade I). It originated in the Central part of Africa ii) WA Clade or West African Clade, the second one from the West African (WA) Clade (Clade II). WA Clade or Clade II has been further divided into Clade IIa and Clade IIb. Nakazawa et al. have highlighted that the branches of the CB clade are much shorter. The researchers indicated a recent diversification within this clade; they have described the biogeographic barriers accountable for the CB-WA split [11]. We have recently developed the phylogenomic illustration of the current MPXV and the mutational landscape [1]. Some other researchers have demonstrated phylogenomic illustration or molecular evolution of the current MPXV [[12], [13], [14]]. At the same time, researchers illustrated two important directions: one, mutation and phylogenetics; second, mutation and pathogenicity [12]. Researchers have found 24 non-synonymous variations. Among them, some mutations such as M1741I, P722S, and D209N, located in B21R surface glycoprotein, are associated with the immune evasion process and enhance transmissibility of the MPXV [15]. Several other researchers found the elevated frequency of TC→TT and GA→AA in the genome sequences [1,15]. However, a continuous mutation is found in the MPXV, which demands a mutation-proof vaccine for the MPXV. Recently, we have already urged for a mutation-proof vaccine for SARS-CoV-2 considering mutations [16]. The mutation-proof vaccine might be more effective against a mutating pathogen including the MPXV. In this case, mutations are considered during the coding sequence selection for vaccine development. The vaccine candidate should be effective, safe, and unable to induce the enriched diseases following infection. Subsequently, large-scale production can be done quickly, increasing the flexibility of vaccines to antigenic changes in circulating strains. Developing next-generation vaccine against MPX is crucial to define our real expectations from this vaccine or the need for the future emerging mutational variants of MPXV.

The current outbreak of MPXV does have certain unfamiliar features, containing the persistent patterns of human-to-human transmission when men have sex with men. Therefore, the human MPXV is no longer considered a sporadic zoonotic disease, and its rapid spread in more than 100 countries has posed high global public health concerns [17]. It also requires more study to know whether any new transmission pattern has emerged or not [18].

We already know about the pandemic periods of SARS-CoV-2 infection, and the virus quickly emerged to become a critical human pathogen causing the ongoing COVID-19 pandemic. The COVID-19 pandemic phases with multiple waves repeatedly faced surge in cases, high mortality, severe infections, breakthrough vaccine infections and reinfection owing to continuously emerging SARS-CoV-2 mutants, variants and lineages possessing higher transmissibility and severe disease causing ability via overpowering protection levels of vaccine induced immunity and antibodies-based therapies through immune escape mechanisms. So, it seems to be proactive to prepare effective and advanced next generation vaccines against any pathogenic viruses or microbes, although they presently may not be showing higher death cases as a major threat. For MPXV, existing vaccines and drugs still have certain shortcomings. Clinical trials have not confirmed the vaccines’ efficacy and safety profile and larger trials are required for assessing smallpox vaccine efficacy against MPX. In this respect, high-end research in advanced platforms is urgently needed to develop suitable next-generation vaccines against MPXV for countering its currently emerging and future threats.

## Ethical approval

No applicable.

## Sources of funding

No fund received.

## Author contribution

**Manojit Bhattacharya:** Data Curation, Investigation, Writing - Original Draft.

**Kuldeep Dhama**: Validation; editing-reviewing.

**Chiranjib Chakraborty:** Conceptualization, Investigation, Writing - Original Draft, Writing - review & editing.

All authors critically reviewed and approved the final version of the manuscript.

## Conflicts of interest

All authors report no conflicts of interest relevant to this article.

## Registration of research studies

1. Name of the registry: Not applicable
2. Unique Identifying number or registration ID: Not applicable
3. Hyperlink to your specific registration (must be publicly accessible and will be checked): Not applicable

## Guarantor

Professor Chiranjib Chakraborty.

Department of Biotechnology, School of Life Science and Biotechnology.

Adamas University, Kolkata, West Bengal 700,126, India.

Email: drchiranjib@yahoo.com Tel: +91-9871608125.

## Consent

Not applicable.

## Provenance and peer review

Not commissioned, internally peer-reviewed.

## Data statement

The data in this correspondence article is not sensitive in nature and is accessible in the public domain. The data is therefore available and not of a confidential nature.
